# Use of Exogenous and Endogenous Photomediators as Efficient ROS Modulation Tools: Results and Perspectives for Therapeutic Purposes

**DOI:** 10.1155/2019/2867516

**Published:** 2019-03-31

**Authors:** Maria Rosa Antognazza, Ilaria Abdel Aziz, Francesco Lodola

**Affiliations:** ^1^Center for Nano Science and Technology, Istituto Italiano di Tecnologia, Via Pascoli 70/3, 20133 Milano, Italy; ^2^Politecnico di Milano, Dipartimento di Fisica, Piazza L. Da Vinci 32, 20133 Milano, Italy

## Abstract

Reactive Oxygen Species (ROS) play an essential dual role in living systems. Healthy levels of ROS modulate several signaling pathways, but at the same time, when they exceed normal physiological amounts, they work in the opposite direction, playing pivotal functions in the pathophysiology of multiple severe medical conditions (i.e., cancer, diabetes, neurodegenerative and cardiovascular diseases, and aging). Therefore, the research for methods to detect their levels via light-sensitive fluorescent probes has been extensively studied over the years. However, this is not the only link between light and ROS. In fact, the modulation of ROS mediated by light has been exploited already for a long time. In this review, we report the state of the art, as well as recent developments, in the field of photostimulation of oxidative stress, from photobiomodulation (PBM) mediated by naturally expressed light-sensitive proteins to the most recent optogenetic approaches, and finally, we describe the main methods of exogenous stimulation, in particular highlighting the new insights based on optically driven ROS modulation mediated by polymeric materials.

## 1. Introduction

For all living aerobic organisms, molecular oxygen is the central compound for cellular respiration, being the ultimate electron acceptor in the biochemical cycle for ATP production. The first reduced state of molecular oxygen, superoxide O_2_^·−^, and all the successive reduced states can be physiologically found in cellular compartments and are called Reactive Oxygen Species (ROS) [1]. The interaction between ROS and fatty acids or nucleic acids leads to the oxidative damage of these compounds [2]; thus, ROS overproduction has been related to many diseases, like age-related and cardiovascular disorders, cancer, and neurodegenerative diseases such as Parkinson's or Alzheimer's [3–6]. This unfortunate correlation is anyway under revision, since ROS are important second messengers for the expression of several transcription factors, regulation of cellular adhesion, redox-mediated amplification of immune response, and programmed cell death [7].

**Figure 1:**
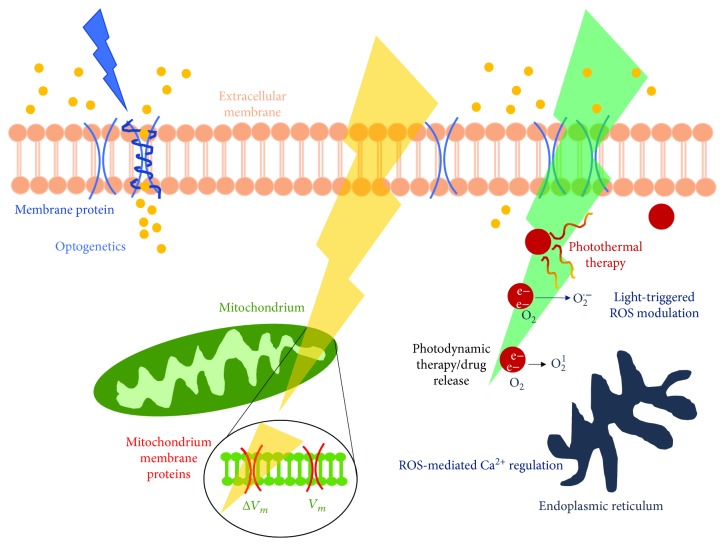
Schematic drawing depicting reviewed approaches for optical modulation of intracellular ROS. Endogenous stimulation (left side of the image) comprises the use of genetically modified light-sensitive proteins and photobiomodulation techniques. Exogenous stimulation techniques addressed here (right side of the image) are based on the use of carbon-based materials and include different approaches, e.g., photodynamic therapy, eventually coupled to optically triggered systems for drug release, and nontoxic ROS photothermal modulation.

Therefore, the need for direct assessment of intracellular ROS concentration fostered the development of probes able to bind oxygen radicals and to selectively detect them, and several fluorescent probes are now commercially available [8]. The conjugation between chemistry and photophysics has emerged as the key to achieve high spatiotemporal resolution and selectivity in ROS detection. A similar approach can be pursued also in the realization of optical actuators, to modulate and finely control the ROS balance at nontoxic levels. However, this route has been investigated to a much lower extent as compared to ROS imaging probes. Still, emerging interest from different research fields, both in biotechnology and materials science, led to promising results. In this review, we will focus the attention on this less beaten path, first describing the control exerted by endogenous photostimulation, from photobiomodulation (PBM) mediated by naturally expressed light-sensitive proteins to optogenetic approaches, followed by describing the main methods to artificially enhance light sensitivity in living cells and thus exogenously stimulate living organism biological activity, in particular highlighting the new insights based on optically driven ROS modulation mediated by polymeric materials. Both approaches are schematically depicted in Figure 1.

## 2. Endogenous Photostimulation: Pros and Cons

The idea to trigger biochemical signals and biological responses by exposing living systems to light is one of the most fascinating insights in science. In fact, optical techniques could be more useful tools rather than the conventional ones based on pharmaceutical and electrical methods because of their higher spatial resolution and possibility to stimulate in a less invasive way. In this section, we will discuss the close link between ROS and photostimulation of endogenous proteins.

First, we will concentrate on PBM, a well-established technique that is able to generate beneficial effects on cells or tissues (i.e., wound healing and tissue regeneration) and can also act as a natural painkiller by generating photochemical reactions by exploiting low-power density lasers or light-emitting diodes.

Below, we will portray one of the newest and at the same time most groundbreaking discoveries for the optical control of cells: optogenetics. Technically, this approach cannot be considered completely endogenous; however, we decided to include it in this category because, even if transfection is necessary in order that cells can express light-sensitive proteins, the biological processes generated by light originate from within the cell itself.

### 2.1. Activation of Naturally Expressed Light-Sensitive Proteins

PBM is the term that describes a technique developed almost 50 years ago by the Hungarian physician Endre Mester exploiting the energy of light to stimulate cells and tissues for therapeutic purposes [9].

For a long time, the technique was also known as “low-level laser therapy,” but since it was discovered that noncoherent light-emitting diodes (LEDs) perform as well as medical lasers, with the advantage of a reduced cost and reduced safety issues [10], it was decided to standardize the nomenclature to PBM [10].

PBM has the advantage of being noninvasive and allows broad application (i.e., pain relief to promote the recovery of tendinopathies, nerve injuries, osteoarthritis, and wound healing) and differs from other light-based assays because it does not cause ablation and it is not based on heating [11]. Although the mechanisms and actual therapeutic perspectives of PBM are still hazy, in recent years, much progress has been made in clarifying the chromophores and signaling pathways involved. The main photobiology dogma states that photons of light must be absorbed by the chromophore located within the tissue to have any biological effect [12]. In the next paragraph, we will discuss the main chromophores involved in PBM.

Since the principal site of absorption in mammalian cells has been identified as the mitochondrion, it is obvious that cells, even those not normally exposed to light during normal living activity, but with a high number of organelles and an intense metabolic activity (i.e., muscle cells, neurons, liver and kidney cells among the others), can be slightly responsive to light [13]. The mitochondrial transmembrane protein cytochrome c oxidase (CCO) is probably the most well-known chromophore. CCO is the last enzyme in the respiratory electron transport chain of the abovementioned organelles converting oxygen into molecular water using the electrons from reduced cytochrome c [14]. Evidence suggests that CCO acts as a photoacceptor and transducer of photosignals in the red and near-infrared (NIR) regions of the light spectrum (620–1000 nm) [14]. PBM acts at this point, inducing a depolarization of the mitochondrial membrane potential (MMP) and increasing the levels of the organic chemical Adenosine Triphosphate (ATP), the second messenger cyclic adenosine monophosphate (cAMP), and ROS as well [15].

Other important classes of photoreceptors that can be directly gated by light are opsins [13]. These transmembrane proteins are part of the light-sensitive G-protein-coupled receptor superfamily and respond particularly to blue (450-495 nm) and green (495-570 nm) light [16]. The most famous opsin is rhodopsin, the primary light-sensitive receptor actuator of vision in the rod and cone photoreceptor cells in the mammalian retina [16]. A direct consequence of light-mediated opsin activation is the opening of light-gated ion channels belonging to the Transient Receptor Potential (TRP) group [17]. The TRP channel superfamily, now classified into seven related subfamilies that are found in most organisms, tissues, and cell types [18], is at the base of the perception of pain, warm and cold temperatures, pressure, and noxious and pungent chemicals and is involved in many different cellular processes. TRP activation causes nonselective permeabilization to ions (typically: calcium, sodium, and magnesium) [19]. Interestingly, it was recently reported that TRP channels are playing a pivotal role in the detection of cellular redox status [20].

One of the most frequently observed changes when PBM experiments are conducted *in vitro* has been the modulation of levels of ROS such as superoxide, hydrogen peroxide, singlet oxygen, and hydroxyl radical [21]. ROS in particular has a Two-Face behavior; while high doses unquestionably exert damage on cellular integrity, at low concentrations, they have beneficial effects by inducing an adaptive response [22].

**Figure 2:**
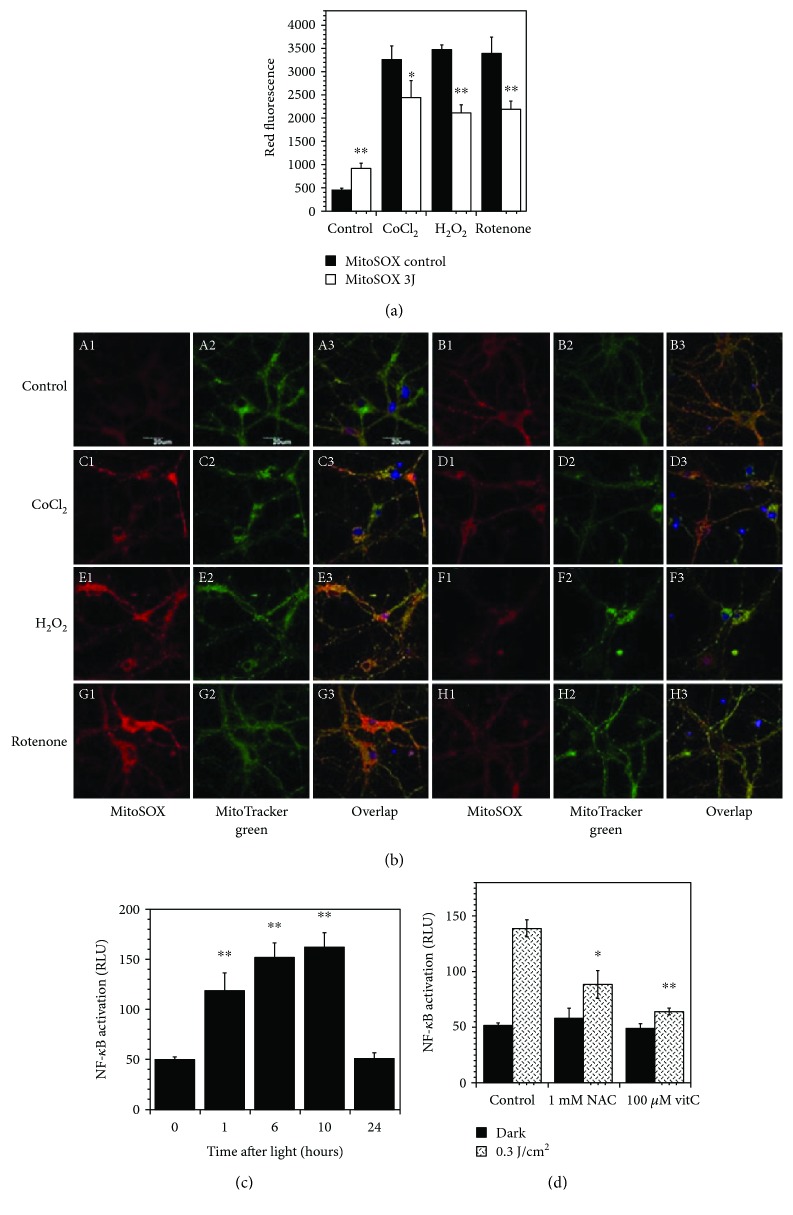
Photobiomodulation (3 J/cm^2^, 810 nm laser) reduces ROS levels and protects cultured cortical neurons from death in oxidatively stressed cells (a). Cortical neurons have been labelled with MitoSOX (red) for mitochondrial, MitoTracker (green) for mitochondrial colocalization, and Hoechst (blue) for nuclei. Subpanels A1–A3 and B1–B3 refer to control cortical neurons in the dark and subjected to the photobiomodulation protocol, respectively; C1–C3 and D1–D3 to neurons treated with CoCl_2_ in the absence and presence of light, respectively; E1–E3 and F1–F3 to neurons treated with H_2_O_2_ in the absence and presence of light, respectively; and G1–G3 and H1–H3 to neurons treated with rotenone in the absence and presence of light, respectively. ROS time course of NF-*κ*B activation after laser irradiation (0.3 J/cm^2^, 810 nm) in mouse embryonic fibroblasts is depicted in (c) while antioxidant therapy with 1 mM N-acetyl cysteine or 100 mM ascorbic acid abrogates laser-induced NF-*κ*B activation (d). The figure is adapted from [23, 28].

In this regard, Huang et al. measured the MMP upon absorption of red/NIR light by mitochondria in normal primary cells. The authors noticed that the MMP increment led to a modest but significant increase in the level of ROS. Interestingly, PBM had an apparent opposite behavior in cells that had already been subjected to oxidative stress [23]. *In vitro* experiments conducted on chemically oxidative stressed cortical neurons evidenced that PBM led to increased levels of MMP (evaluated with tetramethylrhodamine) and ATP and reduced ROS while control cells had a small increase in ROS together with the MMP and ATP levels (Figures 2(a) and 2(b)). This was probably ascribable to an adaptive effect of PBM that tends to increase MMP back towards baseline, thereby lowering ROS production [23].

Not surprisingly, there have been several *in vivo* studies conducted on animal models recapitulating a specific disease or injury in which a reduction in tissue markers of oxidative stress after PBM was measured [24].

Contrarily to red and NIR light stimulation, ROS production by blue light, which is partly responsible for ocular phototoxicity [25], is a topic still less studied, in the framework of PBM. However, preliminary studies in adipose-derived stem cells revealed that illumination with blue light generates an increase in ROS tied to a reduction in ATP levels and MMP and most importantly an impaired cell proliferation [12]. In an interesting way, all these phenomena could be partially blocked by treating cells with capsazepine, a selective inhibitor of TRPV1 channels [26]. However, the detailed relationship of the photoactivation of TRPV1 and ROS generation is still to be completely elucidated and will deserve focused attention in the future.

Finally, it is important to highlight that ROS are able to activate several transcription factors and signaling pathways that could explain why a relatively brief exposure to light can have long-lasting results [27]. For example, in an embryonic fibroblast, a slight increase of ROS was sufficient to lead to the activation of the transcription factor Nuclear Factor Kappa B (NF-*κ*B), a protein complex that is involved in the control of several cellular processes, such as immune and inflammatory responses, growth, and apoptosis, and that can also act as a redox sensor (Figures 2(c) and 2(d)) [28].

### 2.2. Optogenetic Approaches

The definition of “optogenetics” was first coined by Prof. Karl Deisseroth in 2006 to describe the selective control of neuronal activity *in vitro* by light via the expression of genetically targeted photoreceptors [29]. Later, the denotation was extended to both actuators and sensors that are, respectively, proteins which are able to alter the cell activity in which they are expressed after light exposure and genetically encoded, voltage-sensitive fluorescent proteins that can be used to monitor intracellular variations in ionic concentration as well as the amount of extracellular neurotransmitter*s* [30]. Furthermore, novel insights have refined the technique broadening the spectrum of application to systems as complex as live freely moving animals [31]. Optogenetics, as any new technology, has its own drawbacks, but conversely to traditional investigation methods (i.e., direct electrical stimulation and pharmacological intervention) has the undoubted advantage of guaranteeing a high temporal and spatial resolution that does not affect the physiological environment of the system object of the study [32]. Surely, in the prospect of a therapeutic application, the main Achilles' heel is represented by the genetic manipulation dependence of the approach. Thus, demonstrating the long-term safety of gene therapy in patients is mandatory before one can think of applying the technique for clinical purposes.

Conventional methods to study the impact of ROS pathways exploit the global and unselective administration of ROS-generating or ROS-inhibiting chemicals or antioxidants, thus being hardly controllable at the subcell compartment level [33]. Conversely, the use of optogenetic tools may allow to gain unprecedented spatial selectivity, as provided by selective, genetically engineered effector proteins and highly focused optical excitation.

Genetically encoded ROS-generating proteins (RGPs) were originally created for cell ablation and protein inactivation purposes; however, their potential to study ROS signaling cannot be underestimated. The main characteristics that allow RGP to generate a specific biological effect depend on the type of ROS produced and on the compartment where it is produced. Therefore, an intriguing feature of RGPs is their ability to be targeted with high specificity within cell compartments, by taking advantage of commonly used signal sequences (i.e., the SV40 nuclear targeting signal or the TOMM-20 mitochondrial targeting sequence depending on whether you are interested in the nucleus or mitochondria, respectively), thus optimizing RGP impact [34].

KillerRed, the first phototoxic fluorescent protein, is an active version of anm2CP, a homolog of the widely known Green Fluorescent Protein (GFP), a highly exploited commercial tool for imaging approaches mainly considered photochemically inert. Contrariwise, KillerRed under appropriate illumination (red light) is able to generate ROS, in particular O_2_^·−^ via a type I reaction [35]. Notably, since the active form of the protein is a dimer and dimerization can have the drawback of affecting the localization and functionality of the protein, a genetic fusion of two KillerRed coding sequences, called “tandem KillerRed,” was generated to allow intramolecular dimerization and maturation of the protein and successfully exploited to block cell division [36].

Two other newly created RGPs are SuperNova [37] and miniSOG [38]. The first is generated by the random mutagenesis of KillerRed and successfully used to produce both O_2_^·−^ and ^1^O_2_ [37]. The second one, whose acronym stands for singlet oxygen generator, is a small (only 106 amino acids) green fluorescent flavoprotein generated from Arabidopsis phototropin 2 with an excitation maximum at 448 nm [34]. Remarkably, the capability to locally generate reactive ^1^O_2_ has made this RGP an interesting tool for cell ablation [39]. Recently, it was demonstrated that miniSOG monomers can generate O_2_^·−^ rather than ^1^O_2_ [40]_._

However, in addition to those already mentioned above, the number of genetically encoded photosensitizers is growing. For example, Sarkisyan et al. characterized a blue-shifted, orange fluorescent variant of KillerRed [41]. Interestingly, the photoactivation of this new protein, named KillerOrange, does not trigger KillerRed which opens up interesting perspectives for a tandem application of both proteins in a single system. Still, even if it seems that the mechanism of oxidant production is thought to be the same type I (radical) reaction of KillerRed, this new RGP requires further characterization. However, for a more comprehensive and detailed description of the newest fluorescent proteins able to generate oxidants, we recommend a recently published review article by Trewin et al. [42].

The potential application of this approach is remarkable. Going beyond the use of KillerRed in single cells for anticancer therapy [43], it is possible to extend the applicability of genetically encoded photosensitizers to more complex model organisms, in which the introduction or the expression of recombinant exogenous genes is a standard procedure [39, 44–46].

**Figure 3:**
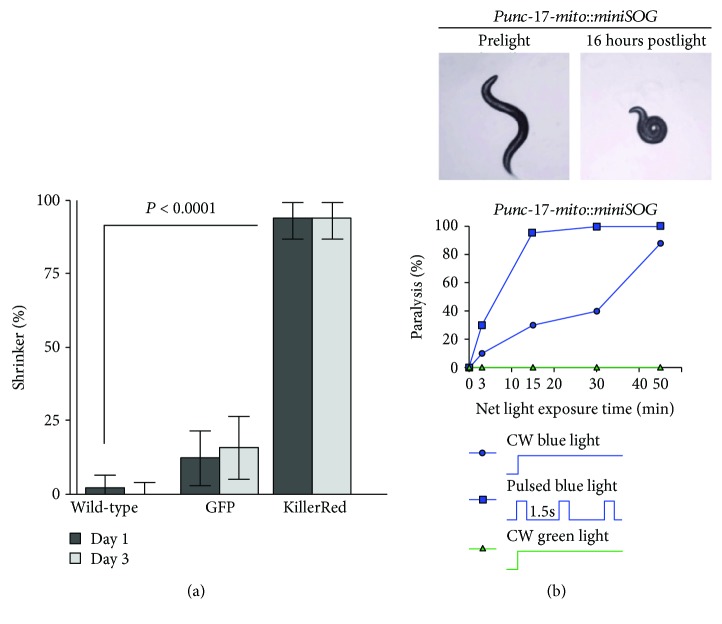
Mitochondrially targeted KillerRed and miniSOG are used as ROS-producing tools for light-induced cell ablation. (a) KillerRed activation in GABAergic neurons causes a shrinker phenotype in *C. elegans* (i.e., longitudinal shortening of the body upon head touch) consistent with the loss of GABAergic neuronal function 24 hours after illumination. (b) MiniSOG causes paralysis in *C. elegans* via ablation of Punc-17*β*-expressing cells. As depicted in the lower panel, light dosage correlates with the percentage of paralysis induced by miniSOG upon illumination under continuous or pulsed light. The figure is adapted from [39, 44].

Photoablation with RGPs was performed in *C. elegans* and both KillerRed (Figure 3(a)) [44] and miniSOG have been used to reach the goal (Figure 3(b)) [38, 39]. However, during photoablation with RGPs, several parameters must be kept in mind. First, the intracellular targeting of an RGP may affect the efficiency of ablation. Notably, mitochondrial targeting generated a higher phototoxic effect and consequent cell death rather than cytosol targeting, due to the fact that the mitochondrion plays a role in mediating cell death [39]. Furthermore, even targeting diverse regions of the mitochondria resulted in different photoablation efficiency, a discrepancy that probably represents the localized ROS buffering capacity [39].

Secondly, phototoxic effects can be modulated by changing the duration and intensity of light stimulation. This was seen in *C. elegans* expressing the outer membrane-targeted miniSOG in motor neurons where varying the light exposure from continuous to pulsed increased the effectiveness of cell ablation [39]. For a future clinical perspective, the development of far-red-shifted variants of RGPs would greatly facilitate their clinical use since for the treatment of tumors *in vivo*, the transparency of the tissue and size of the tumor will become important aspects to be considered. Moreover, Xu and Chisholm recently reported that the membrane-targeted miniSOG can ablate neurons and nonneuronal tissues in *C. elegans* in a most efficient way rather than mito-miniSOG, thus expanding the throughput of optogenetic cell ablation [38].

A complementary approach that exploits ROS for the selective inactivation of a specific protein is chromophore-assisted light inactivation (CALI). CALI, to acutely inactivate a target protein, requires exposing a chromophore to light with an optimized light dose and increased spatial precision. Both KillerRed and miniSOG were successfully used to increase CALI specificity by maintaining the protein of interest close to the photosensitizer [45, 47, 48].

A possible future use of the RGP approach is for the study of ROS signaling that is not easily decipherable for its intrinsic reactive behavior. The possibility to target RGPs to a specific cellular compartment will allow to determine the physiological output of the ROS signal. In addition, the ability to generate specific ROS via different RGPs might provide new hints towards clarification of their peculiar dynamics and physiopathological effects.

Table 1 summarizes the excitation wavelength and types of ROS produced by the native and genetically modified light-sensitive proteins treated in this section.

## 3. Exogenous Photostimulation: The Case of Carbon-Based Materials

ROS optical modulation can be achieved by making use of exogenous transducers, either organic or inorganic. In this review, we will limit our attention only to the first ones. Carbon-based systems, and in particular polymeric materials, have attracted considerable attention in the field of optically driven ROS modulation, mainly due to their enhanced biocompatibility and easier routes for chemical functionalization with molecular moieties and specific drugs, as compared to inorganic compounds.

Considerable efforts have been focused in the latest years in particular on electrically/electrochemically inert polymers, properly endowed with ROS-responsive units for controlled and tuneable drug release [49]. In these cases, light excitation has been primarily used as a stimulus to induce ROS overproduction, usually through a thermally mediated effect. Polymer systems are loaded with specific drugs, whose release is efficiently triggered by the altered, increased level of ROS. Thus, in this case, light is a mere stimulus to increase, in a spatially and temporally controlled manner, ROS levels, and the polymer plays the purely passive role of a biocompatible drug carrier. Based on this approach, several ROS-responsive polymeric micelles have been developed so far; interesting examples include the ROS-responsive triblock copolymer micelles, containing diselenide bonds [50] in a hydrophobic polyurethane block, e.g., poly(ethylene glycol)-b-polyurethane-b-poly(ethylene glycol) (PEG-PUSeSe-PEG) [51].

Following a slightly different approach, interesting light-responsive systems were developed by Han et al. [52], by encapsulating porphyrins as direct photosensitizers in the core of PEG-PUSeSe-PEG micelles. Red light excitation of the photosensitizer determines the production of singlet oxygen, which in turn facilitates the cleavage of the diselenide bond, thus leading to the disruption of the polymer micelle and release of Doxorubicin (DOX). Similar processes were exploited by the realization of NIR light-responsive nanogel systems [53], and anticancer effects were demonstrated *in vitro*. The successful combination of chemo- and photodynamic therapy (PDT) has been also demonstrated through the realization of polymeric micelles based on PEG-b-PCL copolymers endowed with both the photosensitizer and the anticancer drug. Upon visible light excitation, the action of the photosensitizers maximizes the release of the anticancer antibiotic DOX, thus boosting the overall antitumor efficacy [54, 55].

Two-photon excitation (TPE) nanoparticle photosensitizers represent another valuable opportunity for the realization of efficient systems for PDT, since they enable being able to work upon NIR light excitation, thus ensuring deeper tissue penetration. TPE PDT is characterized by the nonlinear absorption of two low-energy photons of NIR light with the resulting emission of higher energy light, in the visible range. The latter sensitizes oxygen to produce ROS at toxic levels, for the treatment of cancer cells. The use of NIR light allows for deeper tissue penetration, to achieve efficient PDT of deep-seated tumors. Recent advances in this field, comprising both organic and inorganic nanoparticles, have been recently reviewed in [56]. Besides notable advantages offered by TPE nanoparticle sensitizers, it is also important to underline that many crucial challenges currently hamper their use in preclinical and clinical practice. First of all, detailed nanotoxicology studies are still lacking. Most of reported systems have been tested only in cell cultures or in mice animal models by intratumoral injection. However, biodistribution, blood circulation, and dark toxicity after systemic administration are largely unknown, especially considering that actual toxicity is a complex function of several parameters (size, surface chemistry, chemical composition, and dose, just to cite some). Moreover, most TPE particles can be also excited by one-photon excitation, thus raising the potential issue of skin phototoxicity following systemic administration. Laser systems for TPE are often complex and expensive systems and do not allow spot sizes and photoexcitation density values suitable for the treatment of deep-seated, bulky tumoral areas. Last but not least, accurate light dosimetry methods suitable for in vivo applications are often unavailable. Despite all these shortcomings and limitations, however, research on TPE nanoparticle sensitizers has made impressive steps forwards, and it is expected that they may represent a suitable choice for precise ROS optical modulation targeted at therapeutic purposes, even beyond their application in photodynamic therapy.

Here, we will limit our attention to carbon-based semiconductors, including carbon dots, nanotubes, and polymers [57, 58]. These are expected to offer enhanced biocompatibility with respect to their inorganic counterparts. Moreover, they usually show higher resistance to photobleaching and larger two-photon absorption cross-sections, thus representing ideal two-photon energy donors. In 2013, a first example of the use of carbon quantum dots (CQD), covalently linked with protoporphyrin IX (PTIX), for TPE PDT was reported by Fowley et al. [59]. Efficient fluorescence resonance energy transfer (FRET) processes between CQD and PTIX and subsequent singlet oxygen generation lead to sizable results both *in vitro* and *in vivo*. *In vitro*, TPE at 800 nm induced a reduction in HeLa cell viability up to 82%. *In vivo*, a fibrosarcoma mouse model was considered, and treatment with CQD-PTIX upon NIR irradiation provoked a 60% shrinkage of the tumor size in 4 days. Conversely, the tumor size of control animals increased by 65%. Further implementation of CQD to achieve higher two-photon absorption cross-sections has been recently reported by Wang et al. [60]. Single-walled carbon nanotubes, loaded with Ru(II) complexes, have been also reported for bimodal photothermal and TPE PDT [61]. This approach seems to be very promising from a therapeutic point of view, for cancer therapy applications. In fact, intratumoral injection of the compound in mice models leads to nearly complete tumor ablation, upon irradiation at 808 nm, with a relative variation in the tumor volume of about -100%, as compared to an increase of about +300% in the control cases. The first example of the use of a polymer endowed with a porphyrin photosystem for TPE dates back to 2007 [62]. A FRET efficiency as high as 96%, leading to substantial singlet oxygen generation, was reported. Initially promising results boosted an intense activity aimed at the optimization of several polymer/photosensitizer compounds for TPE. Some nonexhaustive examples include polyfluorene-, polyphenylene-, and polythiophene-based polymer nanoparticles [57, 58, 63].

Finally, conjugated polymer nanoparticles based on polyphenylenevinylenes have been successfully used as direct PDT sensitizers [64].

Interestingly, a polymer-based system for PDT, endowed with DOX, was recently coupled with a hypoxia-responsive drug-delivery system [65]. Here, the conjugated polymer is used as a visible/NIR light-triggered ROS source, and it is grafted with the hypoxia-sensitive, hydrophobic 2-nitroimidazole (NI). Under hypoxia, NI is reduced to hydrophilic 2-aminoimidazoles, thus allowing the release of the DOX cargo. The *in vivo* efficacy of this approach was tested on HeLa tumor-bearing mice, and almost complete inhibition of the tumor growth was obtained.

Overall, polymer nanoparticles hold the potential to act as versatile, powerful, and biocompatible PDT agents, eventually combined to chemotherapy and hypoxia conditions; however, extensive *in vivo* studies are still lacking, and the reliability and efficacy of this approach remains to be fully confirmed.

In the applications mentioned above, carbon-based materials have been mainly used as passive compounds for controlled drug release, against ROS overproduction, and as active, biocompatible systems for effective photodynamic, anticancer therapy.

Much more recently, the use of organic materials has been also proposed for the active modulation of ROS species at nontoxic levels.

An interesting example has been reported by Miyako et al. [66], based on carbon nanohorns, functionalized with a ROS-generating, NIR light-sensitive dye. As with the other previously mentioned systems, the compound has a twofold function, since, upon NIR excitation, it can generate at the same time both heat and ROS. It was shown that NIR-triggered ROS production, at the level of single cells, leads to a modulation of the Ca^2+^ signaling. Importantly, the viability of the cells treated with the functionalized carbon nanohorns and exposed to NIR excitation was not significantly affected. The nanomodulator was also tested *in vivo*, within a *Xenopus laevis* frog model, and reliable optical modulation of the paw nerve activity was detected.

Very recently, conjugated polymers also started to be considered for nontoxic ROS modulation, thus targeting several other therapeutic applications beyond anticancer therapies.

In this regard, the example of polythiophene derivatives is highly instructive [67–74].

Thiophene-based materials have been extensively used in the field of photovoltaics and photodetection, and in the latest thirty years, they represented workhorse materials for several applications in the optoelectronics field.

**Figure 4:**
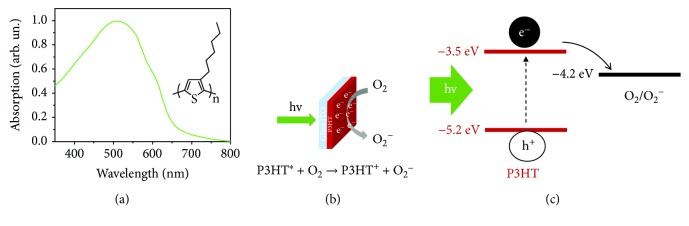
Conjugated polymers based on polythiophene derivatives represent ideal candidates to optically modulate the ROS balance. (a) Regio-regular poly(3-hexyl-thiophene) (rr-P3HT) optical absorption spectrum and chemical structure. (b) Sketch of the electrochemical phenomena occurring at the interface between the polymer thin film surface and an aqueous electrolyte upon visible light excitation. Photoexcitation of the polymer leads to oxygen reduction processes and ROS production. (c) rr-P3HT and oxygen reduction energetic levels.

Conversely, the possibility to use polythiophenes in an aqueous, biological-like environment has been addressed only much more recently [67]. Several works have shown that their main optoelectronic properties are well preserved upon prolonged, direct exposure to water at neutral or acid pH, at variance with other conjugated polymers [75, 76]. Importantly, the biocompatibility of several thiophene derivatives has been largely assessed both *in vitro* and *in vivo* [77, 78]. Our group recently proposed the use of regio-regular poly(3-hexyl-thiophene) (rr-P3HT) as the photoactive component of an artificial prosthesis for sight restoration, entirely based on carbon-based materials. Follow-up of chronical implants has demonstrated the long-term (>9 months) compatibility, stability, and functionality of the device within the biological environment [79]. The rr-P3HT optical absorption spectrum and chemical structure are shown in Figure 4(a).

Interestingly to the present context, organic devices based on rr-P3HT show excellent photocatalytic properties, recently reviewed in [80]. Possible applications include photocatalytic reduction of hydrogen [81, 82] and oxygen in an aqueous electrolyte [68]. In the framework of this work, in particular, the oxygen reduction processes harvest specific interest, being potentially related to the capability to directly modulate the production of ROS in a biological-like environment.

The main photoelectrochemical process occurring at the interface between rr-P3HT and an aqueous electrolyte is schematized in Figure 4(b). We notice that fully similar considerations can be adapted to the case of other conjugated polymers, in particular to low-band gap polymers widely employed in organic photovoltaics.

The energetic levels of rr-P3HT show a good alignment with the reduction potential of the oxygen in neutral conditions, thus satisfying the fundamental condition to efficiently reduce oxygen. Upon optical excitation, charged states are generated within the polymer bulk, which undergo efficient dissociation into free charges, electrons, and holes. At the hybrid solid/liquid interface, electrons react with oxygen dissolved in water and give rise to efficient photoelectrochemical oxygen reduction reactions (Figure 4(c)) [83]. It has been shown that several conjugated polymers, with optical absorption in different regions of the visible spectrum, can serve as suitable photocatalytic materials [84]. The temporal dynamics typical of this process have been experimentally determined, showing that efficient oxygen reduction occurs on a sub-ms timescale [69]. The phenomenon has been also described from a theoretical point of view by making use of a semiclassical approach. In particular, it has been shown that the aqueous solution generates a local polarization of the outermost polymer layers. Upon photoexcitation, the polymer/water is expected to be negatively charged, thus attracting positive ions and perturbing the ion distribution in the aqueous solution [70].

The use of conjugated polymers for direct ROS modulation at nontoxic levels has been explored to a limited extent, despite early promising results.

In a recent work, Hu et al. reported the use of polythiophene modified with dihydropyridine and demonstrated their antioxidative and anti-inflammatory properties in rat aortic endothelial cells [71].

**Figure 5:**
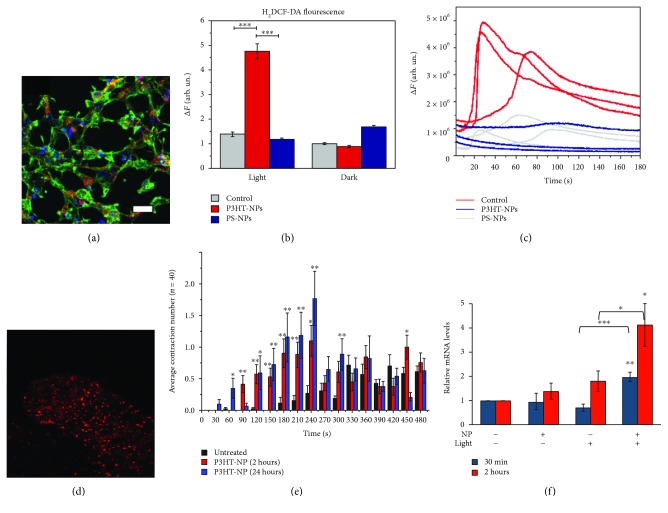
Conjugated polymer NPs are nontoxic optical transducers and modulate cell activity. (a) rr-P3HT NPs are easily internalized within the cell cytosol (stained by phalloidin, green) of HEK-293 cells. Intrinsic NP emission is visible in red; nuclei are stained with DAPI (blue). Scale bar, 30 *μ*m. (b) Photoexcitation of rr-P3HT NPs leads to intracellular ROS production, as evidenced by the variation in the fluorescein diacetate ROS probe fluorescence, in a statistically significant manner as compared to untreated control cells (grey) and cells treated with photoelectrochemically inert nanoparticles of polystyrene (blue). (c) ROS enhancement deterministically triggers intracellular modulation of the Ca^2+^ ion flux, as evidenced by Ca^2+^ imaging experiments. (d) rr-P3HT NPs are internalized within *in vivo* invertebrate animal models of *Hydra vulgaris* without toxicity effects and efficiently modulate the animal behavior. (e) Photoexcitation of polymer beads leads to a sizable increase of animal contraction movements. The average number of tentacle contraction is shown for both untreated and treated animals. A clear variation is observed only during the photoexcitation protocol (shaded grey area) in treated animals. (f) P3HT NP photoexcitation leads to a sizable increase in the expression of opsin3-like genes, as observed by qRT-PCR analysis. Control animals (NPs untreated or treated with NPs but not exposed to visible light excitation) display much lower gene activation. The observation is ascribed to THE optically controlled modulation of ROS levels. The figure is adapted from [72, 74].

In our group, nanoparticles entirely made of rr-P3HT were recently synthesized and extensively tested for their biocompatibility in cell cultures (Figure 5(a)) [85, 86]. In particular, P3HT NPs were administered to Human Embryonic Kidney (HEK-293) cells, efficiently internalized within the cell cytosol and subjected to a photoexcitation protocol (540 nm, 1-100 mW/mm^2^). Interestingly, intracellular generation of ROS was unambiguously attributed to optical stimulation of polymer beads. The only presence of NPs in dark conditions, as well as the optical treatment in the absence of functional, photoelectrochemically active nanomaterials, did not give rise to ROS enhancement (Figure 5(b)). Importantly, P3HT-mediated ROS production does not induce toxic effects on cell viability and physiology, and it deterministically triggers modulation of the intracellular Ca^2+^ ion flux, successfully controlled at the single cell level (Figure 5(c)). In perspective, the capability of polymer NPs to produce ROS and to modulate Ca^2+^ dynamics by illumination on-demand, at nontoxic levels, may open the path to the study of biological processes with a gene-less approach and high spatiotemporal resolution [74].

P3HT nanoparticles were then administered to *in vivo* invertebrate models of *Hydra vulgaris*, without any clear adverse toxicity effect (Figure 5(d)). Interestingly, it was shown that P3HT nanoparticles are able to enhance the animal light sensitivity, inducing a precise behavioral response and enhancing the expression levels of a gene involved in phototransduction, the opsin3-like gene (Figures 5(e) and 5(f)) [72].

Importantly, we have shown that P3HT nanoparticles, once internalized within the animal body and exposed to visible light excitation, produce an increase of oxidative stress parameters without, however, signs of toxicity. Animal antioxidant defense mechanisms are modulated as a result of polymer-mediated photoexcitation, and a clear effect on intracellular redox balance is observed [73].

Though preliminary, these results suggest the possibility to further exploit the use of polymer photocatalytic materials for selective, spatially and temporally controlled, on-demand ROS modulation. The opportunity to use different photoexcitation intensities, stimulation frequencies, light patterns, and materials doses will open the way to potential application either to provoke localized cell damage or hopefully to balance ROS levels and restore physiological conditions in the case of dysfunctions.

Table 2 provides a nonexhaustive list of some cited representative examples of exogenous photomediators for the modulation of ROS levels. Beyond the largely common use of continuous wave vis/NIR light as the activating agent, reported systems deeply differ under most other aspects (e.g., molecular composition, dimensions, photoexcitation density, stimulation temporal protocols, and adopted biological models). Thus, a comparative evaluation among different systems is not possible at the moment. Nevertheless, the variety of organic photomediators for ROS modulation demonstrates the high development potential for different therapeutic applications.

## 4. Current Issues and Future Perspectives

In this manuscript, we have provided a brief overview of currently available techniques to optically modulate the ROS intracellular balance. All of them are characterized by a number of advantages and drawbacks.

The exploitation of endogenous absorption has many benefits; PBM is a cross-sectoral approach that can be applied to a vast range of different biological systems (i.e., from the simplest cell model up to clinical studies). However, its outcome is often weak and leads to somewhat contradictory results, depending on the specific optical protocols. Effects on cell metabolism may critically depend on light frequency, excitation density, and wavelengths, as well as on target biological sites. Unfortunately, the available experimental data are still partial, and the details of photoexcitation are not always provided with the necessary standardization. Thus, further studies will certainly allow to better identify physiological pathways, to achieve a more repeatable ROS modulation, and, ultimately, to additionally ameliorate the therapeutic potential of this technique.

Optogenetic tools hold the promise to enable ROS generation control with high efficiency and unprecedented spatiotemporal resolution, at the level of cell organelles, in a highly selective and repeatable way. Furthermore, new variants of more selective and performing genetically encoded photosensitizers are constantly produced. This, in combination with the advances in lighting, will continue to make genetically encoded photosensitizers pivotal to the advancement of redox biology. Nevertheless, it is important to underline that therapeutic applicability in human subjects does not seem to be in reach at the moment, due to safety issues related to the need for viral transfection.

Finally, we believe that the use of conjugated polymers, as exogenous actuators, may represent an interesting opportunity for an on-demand, carefully controlled ROS modulation. In perspective, they may offer a number of peculiar advantages: (i) the direct responsivity of the active material to visible and NIR light; (ii) the excellent matching with oxygen reduction potential; (iii) superior mechanical properties and straightforward fabrication technology; (iv) the possibility to optically modulate ROS production with high spatial resolution, within the limits of visible light diffraction (~500 nm), thus allowing to target subcellular compartments without making recourse to viral transfer; (v) the opportunity to temporally trigger the on-demand ROS production, by properly patterning the photoexcitation protocol; and (vi) the availability of several strategies to modulate the overall ROS modulation efficiency at toxic or safe levels (e.g., by chemical functionalization of the exogenous organic actuator with proper ROS catalyst/inhibiting agents and drugs and/or by careful tuning of the light excitation density). Despite all these pros, conjugated polymers also put a number of cons, mainly related to the synthesis of photoelectrochemically stable materials suitable for chronic use within the harsh biological environment. In addition, selective targeting of cell subcompartments may be not straightforward.

The careful and selective modulation of ROS appears to be key for future therapeutic perspectives. This ambitious goal will only be achieved by combining interdisciplinary knowledge, from optics to physiology, up to materials science and biomedical engineering, thus making oxidative and regenerative medicine ready for the bedside.

## Figures and Tables

**Table 1:** ROS modulation by endogenous photomediators—some representative examples.

Endogenous photomediator system	ROS-generating protein	Excitation wavelength (nm)	Photoexcitation density	Light stimuli duration	Biological model	Refs
Photobiomodulation	Cytochrome c oxidase	810	20 mW/cm^2^	150 sec	Cortical neurons	[23]

Optogenetics	KillerRed	540-580	0.5 W/cm^2^	30 sec	HeLa cells	[36]
	tdKillerRed	540-580	5.1 mW/mm^2^	5 min	*C. elegans*	[44]
	SuperNova	579	8 W/cm^2^	90 sec	HeLa cells	[37]
	MiniSOG	460	2 mW/cm^2^	12 min	*C. elegans*	[38]
		475	57 mW/cm^2^	0.5 sec on/1.5 sec off	*C. elegans*	[39]
	KillerOrange	512	1 W/cm^2^	60 sec	*E. coli*/HEK-293 cells	[41]

**Table 2:** ROS modulation by exogenous photomediators—some representative examples.

Exogenous photomediator system	Technical approach	Excitation wavelength (nm)	Photoexcitation density	Concentration	Light stimuli duration	Biological model	Refs
Porphyrins	Photosensitizers for drug release	600-780	—	0.001-0.1 mg/mL	Hours	Human hepatic cell lines L-02	[52]
Polymeric micelle PEG-b-PCL	Combination of chemo- and photodynamic therapy	660	50 mW/cm^2^	0.01-5 *μ*g/mL	Hours	PC3 and HeLa cell lines	[55]
Carbon quantum dots linked with protoporphyrin IX	Two-photon excitation PDT	800	~5 mW/cm^2^	0.1-2 *μ*M	1 hour	HeLa cells	[56]
		800	—	30 *μ*M	3 min light/3 min dark	Fibrosarcoma mouse model	[56]
Carbon nanotubes loaded with Ru(II) complex	Photothermal and TPE PDT	808	0.25 W/cm^2^	10-200 *μ*g/mL	5 min	HeLa cells/HeLa tumor mice models	[61]
Polymer/photosensitizer compounds	TPE PDT	White light (400-800)	2-8 mW/cm^2^	1-10 *μ*M	30 min	MCF-7 cancer cells	[63]
Carbon nanohorns	Photothermal therapy/nontoxic ROS modulation	808	104 *μ*W/*μ*m^2^	12.5 *μ*g/mL	3 min	ND7/23 cells/RAW264.7	[66]
		800	292 mW/mm^2^	300 *μ*g/mL	/	*Xenopus laevis*	[66]
Polythiophene beads	Nontoxic ROS modulation	540	1-100 mW/mm^2^	~10 *μ*M	2 min	HEK-293 cells	[73]
		White light (400-800)	0.4 mW/mm^2^	0.1-10 *μ*M	3-30 min	*Hydra vulgaris*	[75]

## References

[1] Krumova K., Cosa G., Nonell S., Flors C. (2016). Chapter 1. Overview of reactive oxygen species. *Comprehensive Series in Photochemical & Photobiological Sciences0*.

[2] Halliwell B. (2006). Reactive species and antioxidants. Redox biology is a fundamental theme of aerobic life. *Plant Physiology*.

[3] Dickinson B. C., Chang C. J. (2011). Chemistry and biology of reactive oxygen species in signaling or stress responses. *Nature Chemical Biology*.

[4] Dröge W. (2002). Free radicals in the physiological control of cell function. *Physiological Reviews*.

[5] Barnham K. J., Masters C. L., Bush A. I. (2004). Neurodegenerative diseases and oxidative stress. *Nature Reviews Drug Discovery*.

[6] Finkel T., Serrano M., Blasco M. A. (2007). The common biology of cancer and ageing. *Nature*.

[7] Winterbourn C. C. (2008). Reconciling the chemistry and biology of reactive oxygen species. *Nature Chemical Biology*.

[8] Gomes A., Fernandes E., Lima J. L. F. C. (2005). Fluorescence probes used for detection of reactive oxygen species. *Journal of Biochemical and Biophysical Methods*.

[9] Mester A., Mester A. (2017). The history of photobiomodulation: Endre Mester (1903–1984). *Photomedicine and Laser Surgery*.

[10] de Freitas L. F., Hamblin M. R. (2016). Proposed mechanisms of photobiomodulation or low-level light therapy. *IEEE Journal of Selected Topics in Quantum Electronics*.

[11] Saltmarche A. E., Naeser M. A., Ho K. F., Hamblin M. R., Lim L. (2017). Significant improvement in cognition in mild to moderately severe dementia cases treated with transcranial plus intranasal photobiomodulation: case series report. *Photomedicine and Laser Surgery*.

[12] Hamblin M. R. (2018). Mechanisms and mitochondrial redox signaling in photobiomodulation. *Photochemistry and Photobiology*.

[13] Hamblin M. R. (2017). Mechanisms and applications of the anti-inflammatory effects of photobiomodulation. *AIMS Biophysics*.

[14] Mason M. G., Nicholls P., Cooper C. E. (2014). Re-evaluation of the near infrared spectra of mitochondrial cytochrome *c* oxidase: implications for non invasive in vivo monitoring of tissues. *Biochimica et Biophysica Acta (BBA) - Bioenergetics*.

[15] Wu S., Zhou F., Wei Y., Chen W. R., Chen Q., Xing D. (2014). Cancer phototherapy via selective photoinactivation of respiratory chain oxidase to trigger a fatal superoxide anion burst. *Antioxidants & Redox Signaling*.

[16] Shichida Y., Matsuyama T. (2009). Evolution of opsins and phototransduction. *Philosophical Transactions of the Royal Society B: Biological Sciences*.

[17] Poletini M. O., Moraes M. N., Ramos B. C., Jerônimo R., Castrucci A. M. d. L. (2015). TRP channels: a missing bond in the entrainment mechanism of peripheral clocks throughout evolution. *Temperature*.

[18] Ramsey I. S., Delling M., Clapham D. E. (2006). An introduction to TRP channels. *Annual Review of Physiology*.

[19] Talavera K., Nilius B., Voets T. (2008). Neuronal TRP channels: thermometers, pathfinders and life-savers. *Trends in Neurosciences*.

[20] Ogawa N., Kurokawa T., Mori Y. (2016). Sensing of redox status by TRP channels. *Cell Calcium*.

[21] Chen A. C.-H., Huang Y.-Y., Arany P. R., Hamblin M. R. Role of reactive oxygen species in low level light therapy.

[22] Popa-Wagner A., Mitran S., Sivanesan S., Chang E., Buga A.-M. (2013). ROS and brain diseases: the good, the bad, and the ugly. *Oxidative Medicine and Cellular Longevity*.

[23] Huang Y.-Y., Nagata K., Tedford C. E., McCarthy T., Hamblin M. R. (2013). Low-level laser therapy (LLLT) reduces oxidative stress in primary cortical neurons in vitro. *Journal of Biophotonics*.

[24] Tatmatsu-Rocha J. C., Ferraresi C., Hamblin M. R. (2016). Low-level laser therapy (904 nm) can increase collagen and reduce oxidative and nitrosative stress in diabetic wounded mouse skin. *Journal of Photochemistry and Photobiology B: Biology*.

[25] Shang Y.-M., Wang G.-S., Sliney D. H., Yang C.-H., Lee L.-L. (2017). Light-emitting-diode induced retinal damage and its wavelength dependency in vivo. *International Journal of Ophthalmology*.

[26] Lodola F., Martino N., Tullii G., Lanzani G., Antognazza M. R. (2017). Conjugated polymers mediate effective activation of the mammalian ion channel transient receptor potential vanilloid 1. *Scientific Reports*.

[27] Kohlgrüber S., Upadhye A., Dyballa-Rukes N., McNamara C. A., Altschmied J. (2017). Regulation of transcription factors by reactive oxygen species and nitric oxide in vascular physiology and pathology. *Antioxidants & Redox Signaling*.

[28] Chen A. C.-H., Arany P. R., Huang Y.-Y. (2011). Low-level laser therapy activates NF-kB via generation of reactive oxygen species in mouse embryonic fibroblasts. *PLoS One*.

[29] Deisseroth K., Feng G., Majewska A. K., Miesenbock G., Ting A., Schnitzer M. J. (2006). Next-generation optical technologies for illuminating genetically targeted brain circuits. *Journal of Neuroscience*.

[30] Dugué G. P., Akemann W., Knöpfel T. (2012). A comprehensive concept of optogenetics. *Progress in Brain Research*.

[31] Kawazoe Y., Yawo H., Kimura K. D. (2013). A simple optogenetic system for behavioral analysis of freely moving small animals. *Neuroscience Research*.

[32] Boyden E. (2011). A history of optogenetics: the development of tools for controlling brain circuits with light. *F1000 Biology Reports*.

[33] Dikalov S. I., Harrison D. G. (2014). Methods for detection of mitochondrial and cellular reactive oxygen species. *Antioxidants & Redox Signaling*.

[34] Wojtovich A. P., Foster T. H. (2014). Optogenetic control of ROS production. *Redox Biology*.

[35] Liao Z.-X., Li Y.-C., Lu H.-M., Sung H.-W. (2014). A genetically-encoded KillerRed protein as an intrinsically generated photosensitizer for photodynamic therapy. *Biomaterials*.

[36] Serebrovskaya E. O., Gorodnicheva T. V., Ermakova G. V. (2011). Light-induced blockage of cell division with a chromatin-targeted phototoxic fluorescent protein. *The Biochemical Journal*.

[37] Takemoto K., Matsuda T., Sakai N. (2013). SuperNova, a monomeric photosensitizing fluorescent protein for chromophore-assisted light inactivation. *Scientific Reports*.

[38] Xu S., Chisholm A. D. (2016). Highly efficient optogenetic cell ablation in *C. elegans* using membrane-targeted miniSOG. *Scientific Reports*.

[39] Qi Y. B., Garren E. J., Shu X., Tsien R. Y., Jin Y. (2012). Photo-inducible cell ablation in *Caenorhabditis elegans* using the genetically encoded singlet oxygen generating protein miniSOG. *Proceedings of the National Academy of Sciences of the United States of America*.

[40] Barnett M. E., Baran T. M., Foster T. H., Wojtovich A. P. (2018). Quantification of light-induced miniSOG superoxide production using the selective marker, 2-hydroxyethidium. *Free Radical Biology & Medicine*.

[41] Sarkisyan K. S., Zlobovskaya O. A., Gorbachev D. A. (2015). KillerOrange, a genetically encoded photosensitizer activated by blue and green light. *PLoS One*.

[42] Trewin A. J., Berry B. J., Wei A. Y., Bahr L. L., Foster T. H., Wojtovich A. P. (2018). Light-induced oxidant production by fluorescent proteins. *Free Radical Biology & Medicine*.

[43] Serebrovskaya E. O., Edelweiss E. F., Stremovskiy O. A., Lukyanov K. A., Chudakov D. M., Deyev S. M. (2009). Targeting cancer cells by using an antireceptor antibody-photosensitizer fusion protein. *Proceedings of the National Academy of Sciences of the United States of America*.

[44] Williams D. C., el Bejjani R., Ramirez P. M. (2013). Rapid and permanent neuronal inactivation in vivo via subcellular generation of reactive oxygen with the use of KillerRed. *Cell Reports*.

[45] Bulina M. E., Chudakov D. M., Britanova O. V. (2006). A genetically encoded photosensitizer. *Nature Biotechnology*.

[46] Teh C., Chudakov D. M., Poon K.-L. (2010). Optogenetic in vivo cell manipulation in KillerRed-expressing zebrafish transgenics. *BMC Developmental Biology*.

[47] Bulina M. E., Lukyanov K. A., Britanova O. V., Onichtchouk D., Lukyanov S., Chudakov D. M. (2006). Chromophore-assisted light inactivation (CALI) using the phototoxic fluorescent protein KillerRed. *Nature Protocols*.

[48] Lin J. Y., Sann S. B., Zhou K. (2013). Optogenetic inhibition of synaptic release with chromophore-assisted light inactivation (CALI). *Neuron*.

[49] Saravanakumar G., Kim J., Kim W. J. (2017). Reactive-oxygen-species-responsive drug delivery systems: promises and challenges. *Advanced Science*.

[50] Manjare S. T., Kim Y., Churchill D. G. (2014). Selenium- and tellurium-containing fluorescent molecular probes for the detection of biologically important analytes. *Accounts of Chemical Research*.

[51] Ma N., Li Y., Xu H., Wang Z., Zhang X. (2010). Dual redox responsive assemblies formed from diselenide block copolymers. *Journal of the American Chemical Society*.

[52] Han P., Li S., Cao W. (2013). Red light responsive diselenide-containing block copolymer micelles. *Journal of Materials Chemistry B*.

[53] Tian Y., Zheng J., Tang X., Ren Q., Wang Y., Yang W. (2015). Near-infrared light-responsive nanogels with diselenide-cross-linkers for on-demand degradation and triggered drug release. *Particle & Particle Systems Characterization*.

[54] Baugh S. D. P., Yang Z., Leung D. K., Wilson D. M., Breslow R. (2001). Cyclodextrin dimers as cleavable carriers of photodynamic sensitizers. *Journal of the American Chemical Society*.

[55] Saravanakumar G., Lee J., Kim J., Kim W. J. (2015). Visible light-induced singlet oxygen-mediated intracellular disassembly of polymeric micelles co-loaded with a photosensitizer and an anticancer drug for enhanced photodynamic therapy. *Chemical Communications*.

[56] Shen Y., Shuhendler A. J., Ye D., Xu J.-J., Chen H.-Y. (2016). Two-photon excitation nanoparticles for photodynamic therapy. *Chemical Society Reviews*.

[57] Grimland J. L., Wu C., Ramoutar R. R., Brumaghim J. L., McNeill J. (2011). Photosensitizer-doped conjugated polymer nanoparticles with high cross-sections for one- and two-photon excitation. *Nanoscale*.

[58] Shen X., He F., Wu J., Xu G. Q., Yao S. Q., Xu Q.-H. (2011). Enhanced two-photon singlet oxygen generation by photosensitizer-doped conjugated polymer nanoparticles. *Langmuir*.

[59] Fowley C., Nomikou N., McHale A. P., McCaughan B., Callan J. F. (2013). Extending the tissue penetration capability of conventional photosensitisers: a carbon quantum dot–protoporphyrin IX conjugate for use in two-photon excited photodynamic therapy. *Chemical Communications*.

[60] Wang J., Zhang Z., Zha S. (2014). Carbon nanodots featuring efficient FRET for two-photon photodynamic cancer therapy with a low fs laser power density. *Biomaterials*.

[61] Zhang P., Huang H., Huang J. (2015). Noncovalent ruthenium(II) complexes–single-walled carbon nanotube composites for bimodal photothermal and photodynamic therapy with near-infrared irradiation. *ACS Applied Materials & Interfaces*.

[62] Chen C.-Y., Tian Y., Cheng Y.-J., Young A. C., Ka J.-W., Jen A. K.-Y. (2007). Two-photon absorbing block copolymer as a nanocarrier for porphyrin: energy transfer and singlet oxygen generation in micellar aqueous solution. *Journal of the American Chemical Society*.

[63] Wang B., Yuan H., Zhu C. (2012). Polymer-drug conjugates for intracellar molecule-targeted photoinduced inactivation of protein and growth inhibition of cancer cells. *Scientific Reports*.

[64] Gesquiere A., Jasim K., Topps M. (2018). Conjugated polymer nanotherapeutics for next generation photodynamic therapy. *Medical Research Archives*.

[65] Qian C., Yu J., Chen Y. (2016). Light-activated hypoxia-responsive nanocarriers for enhanced anticancer therapy. *Advanced Materials*.

[66] Miyako E., Russier J., Mauro M. (2014). Photofunctional nanomodulators for bioexcitation. *Angewandte Chemie International Edition*.

[67] Bellani S., Fazzi D., Bruno P. (2014). Reversible P3HT/oxygen charge transfer complex identification in thin films exposed to direct contact with water. *The Journal of Physical Chemistry C*.

[68] Bellani S., Ghadirzadeh A., Meda L. (2015). Hybrid organic/inorganic nanostructures for highly sensitive photoelectrochemical detection of dissolved oxygen in aqueous media. *Advanced Functional Materials*.

[69] Tullii G., Desii A., Bossio C. (2017). Bimodal functioning of a mesoporous, light sensitive polymer/electrolyte interface. *Organic Electronics*.

[70] Mosconi E., Salvatori P., Saba M. I. (2016). Surface polarization drives photoinduced charge separation at the P3HT/water interface. *ACS Energy Letters*.

[71] Hu R., Li S.-L., Bai H.-T. (2016). Regulation of oxidative stress inside living cells through polythiophene derivatives. *Chinese Chemical Letters*.

[72] Tortiglione C., Antognazza M. R., Tino A. (2017). Semiconducting polymers are light nanotransducers in eyeless animals. *Science Advances*.

[73] Moros M., Lewinska A., Onorato G. (2018). Light-triggered modulation of cell antioxidant defense by polymer semiconducting nanoparticles in a model organism. *MRS Communications*.

[74] Bossio C., Abdel Aziz I., Tullii G. (2018). Photocatalytic activity of polymer nanoparticles modulates intracellular calcium dynamics and reactive oxygen species in HEK-293 cells. *Frontiers in Bioengineering and Biotechnology*.

[75] Bellani S., Porro M., Caddeo C. (2015). The study of polythiophene/water interfaces by sum-frequency generation spectroscopy and molecular dynamics simulations. *Journal of Materials Chemistry B*.

[76] Antognazza M. R., Ghezzi D., Musitelli D., Garbugli M., Lanzani G. (2009). A hybrid solid-liquid polymer photodiode for the bioenvironment. *Applied Physics Letters*.

[77] Vaquero S., Bossio C., Bellani S. (2016). Conjugated polymers for the optical control of the electrical activity of living cells. *Journal of Materials Chemistry B*.

[78] Antognazza M. R., Di Paolo M., Ghezzi D. (2016). Characterization of a polymer-based, fully organic prosthesis for implantation into the subretinal space of the rat. *Advanced Healthcare Materials*.

[79] Maya-Vetencourt J. F., Ghezzi D., Antognazza M. R. (2017). A fully organic retinal prosthesis restores vision in a rat model of degenerative blindness. *Nature Materials*.

[80] Bellani S., Antognazza M. R., Bonaccorso F. (2018). Carbon-based photocathode materials for solar hydrogen production. *Advanced Materials*.

[81] Fumagalli F., Bellani S., Schreier M. (2016). Hybrid organic–inorganic H_2_-evolving photocathodes: understanding the route towards high performance organic photoelectrochemical water splitting. *Journal of Materials Chemistry A*.

[82] Rojas H. C., Bellani S., Fumagalli F. (2016). Polymer-based photocathodes with a solution-processable cuprous iodide anode layer and a polyethyleneimine protective coating. *Energy & Environmental Science*.

[83] Suppes G., Ballard E., Holdcroft S. (2013). Aqueous photocathode activity of regioregular poly(3-hexylthiophene). *Polymer Chemistry*.

[84] Lanzarini E., Antognazza M. R., Biso M. (2012). Polymer-based photocatalytic hydrogen generation. *The Journal of Physical Chemistry C*.

[85] Zucchetti E., Zangoli M., Bargigia I. (2017). Poly(3-hexylthiophene) nanoparticles for biophotonics: study of the mutual interaction with living cells. *Journal of Materials Chemistry B*.

[86] Di Maria F., Lodola F., Zucchetti E., Benfenati F., Lanzani G. (2018). The evolution of artificial light actuators in living systems: from planar to nanostructured interfaces. *Chemical Society Reviews*.

